# Influence of in utero fetal death on perineal tears in vaginal deliveries

**DOI:** 10.1038/s41598-023-34185-w

**Published:** 2023-05-09

**Authors:** Thibaud Boudry, Marine Lallemant, Rajeev Ramanah, Nicolas Mottet

**Affiliations:** 1 Department of Obstetrics and Gynecology, University Medical Center of Besancon, University of Franche-Comte, Alexander Fleming Boulevard, 25000 Besançon, France; 2 Université de Franche-Comté, FEMTO-ST Institute, UMR CNRS 6174, Department of Applied Mechanics, Besançon, France; 3 Nanomedicine Laboratory, INSERM EA4662, University of Franche-Comte, 25000 Besancon, France

**Keywords:** Urinary tract, Anatomy, Physics

## Abstract

The aim of this work was to evaluate and compare the incidence of perineal tears and Obstetrical anal sphincter injuries (OASIS) after vaginal delivery following a in utero fetal death (IUFD) compared with those with a live-birth. We conducted a single-center, retrospective cohort study using a database of all women who underwent a spontaneous vaginal delivery in the level III maternity ward. Exclusion criteria were breech presentation, cesarean section birth, instrumental delivery, multiple pregnancy, delivery before 24 + 6 weeks of gestation (WG) and termination of pregnancy for medical reasons. Women from the database were divided into two groups: an "in utero fetal death" (IUFD) group and a control group. Women were included in the IUFD group if they had a spontaneous vaginal delivery following a fetal demise after 24 + 6 WG in cephalic presentation between January 2006 and June 2020. Women in the "control" group were selected from the same database and were included if they underwent a spontaneous vaginal delivery of a live fetus in cephalic presentation, after 24 + 6 WG, during the same period. Each woman in the "IUFD" group was matched to two women (ratio 1:2) in the control group for parity, maternal age, body mass index, gestation and birth weight. The primary outcome was the presence of a sutured or unsutured perineal tear. During the study period, 31,208 patients delivered at a level III maternity hospital. Among them, 215 and 430 women were included in the IUFD group and the control group respectively. The two groups were comparable for all demographic and clinical characteristics except for an epidural analgesia (92% versus 70% in the control group, p < 0.01) and labor induction (86% versus 17% in the control group, p < 0.01). The incidence of any perineal tears was 13% (28/15) in the IUFD group versus 16% (70/430) in the control group. Relative risk of any perineal tears was non significative (RR = 0.8 IC95% [0.5–1.2]). The incidence of first-degree perineal tears was 10% in the IUFD group and 11% in the control group. The incidence of second-degree perineal tears was 18% in the IUFD group and 28% in the control group. Relative risk of first-degree perineal tears (RR = 0.88 95% CI [0.5–1.4]) and second-degree tears (RR = 0.51 95% CI [0.2–1.4]) were non significative. No obstetrical anal sphincter injury was found in either group. Vaginal delivery following a fetal demise did not appear to be either a risk factor or a protective factor for perineal tears. But there as a trend toward a lower incidence of second degree perineal tears in this context.

## Introduction

The incidence of obstetric anal sphincter injuries (OASIS) during spontaneous vaginal delivery is between 0.25 and 6% in France for all women, and between 1.4 and 16% in primiparous women, according to several studies^1–3^.

In the literature, OASIS risk factors are parity, instrumental delivery, prolonged second stage of labor, neonatal birth weight and the head position^4^. OASIS can lead to significant morbidity such as anal incontinence, vulvodynia, perineal pain and dyspareunia^5^. Prevention of these injuries are a priority in labor room. At the time of birth, perineal protection such as slowing and controlling the head expulsion, supporting the perineum, using warm compress or performing Couder's maneuver can reduce the risk of perineal tears^6^.

In France, in utero fetal death (IUFD) is an uncommon pathology that complicates 0.5% of pregnancies^7^. In this case, vaginal deliveries are mostly performed because of a lower risk to the mother than a caesarean delivery. The incidence of OASIS during vaginal delivery of a stillborn child remains unknown. In the literature, biomechanics data of a vaginal delivery following a fetal demise are lacking.

The hypothesis of our work was that the anal sphincter injuries rate during vaginal delivery following a fetal demise would be lower than during a spontaneous vaginal delivery of a liveborn child. Indeed, the perineal deformation could be less important because of lesser perineal mechanical constraints related to the absence of fetal tone and to the maceration of the fetal tissues.

The objective of this study was to evaluate and compare the incidence of perineal tears and OASIS after vaginal delivery following a fetal stillbirth compared with those with a live birth.

## Material and methods

We conducted a single-center, retrospective cohort study using a database of all women who underwent a spontaneous vaginal delivery in the level III maternity ward of Besançon University Hospital. Exclusion criteria were podalic presentation, cesarean section birth, multiple pregnancy, delivery before 24 + 6 weeks of gestation (WG) termination of pregnancy for medical reasons. Instrumental deliveries were also excluded because our team uses mainly the vacuum in live birth and spatulas or forceps in fetal still birth. The groups would not have been comparable. Women from the database were divided into two groups: an " in utero fetal death" (IUFD) group and a "control" group. In utero fetal death was defined as a fetal loss after 24 + 6 WG. Women were included in the “in utero fetal death” (IUFD) group if they had a spontaneous vaginal delivery following a fetal demise in cephalic presentation at the level 3 maternity hospital of Besançon between January 2006 and June 2020. Women in the "control" group were selected from the same database and were included if they underwent a spontaneous vaginal delivery of a live fetus in cephalic presentation, after 24 + 6 WG, during the same period.

Each woman in the "IUFD" group was matched to two women (ratio 1:2) in the control group for parity (exact match), maternal age (age group: < 20, 21–25, 26–30,31–35, 35–40, > 40 years), body mass index (BMI) at the beginning of pregnancy (BMI group used: < 25, 25–35, > 35 kg/m^2^), gestation (identical ± 1 week) and birth weight (identical ± 100 g). These five variables were given equal priority.

In case of IUFD, labor induction was performed after the diagnosis of fetal demise with Mifepristone, Misoprostol, Foley catheter, artificial rupture of membranes and oxytocin according to the gestation, the Bishop’s score and the history of scarred uterus.

All data were anonymized for analysis. Data collected were maternal, obstetric and neonatal characteristics. The primary outcome was the presence of a sutured or unsutured perineal tear. Perineal tears were defined as first, second, third or fourth degree according to the RCOG classification^8^. Obstetrical anal sphincter injury (OASI) was defined as damage of the sphincter complex and/or the anal mucosa. Perineal tears were diagnosed by the midwife or the obstetrician who performed the delivery. In case of a second degree or more severe perineal tears, a double clinical checking by the midwife and the obstetrician was systematically executed.

Relative risk ratios with 95% confidence intervals were calculated for the incidence of any perineal tears and for each degree in the two groups. Demographic and clinical data were compared using a student’s t-test for continuous data. For nonparametric data, a Mann–Whitney test was used. A Fisher test compared categorical variables. A "p" less than 0.05 was used to reject the null hypothesis.

According to French regulations, our study was exempt from ethics committee approval since this observational study used anonymized data from a medical database.

In our center, and so, in this study, women were systematically informed and gave consent that their data could be used for practice evaluation purposes. If not, they were all explicitly informed of the possibility to sign a refusal document. This study complies with to the reference MR004 published by the French Commission of liberties and computer science (= Commission des libertés et de l’informatique or CNIL). The clinical research and innovation department (DRCI) of the Hospital of Besançon approved this study.

All patients were informed and gave their informed consent for the use of their data. All methods were performed in accordance with the relevant guidelines and regulations.

## Results

**Figure 1:**
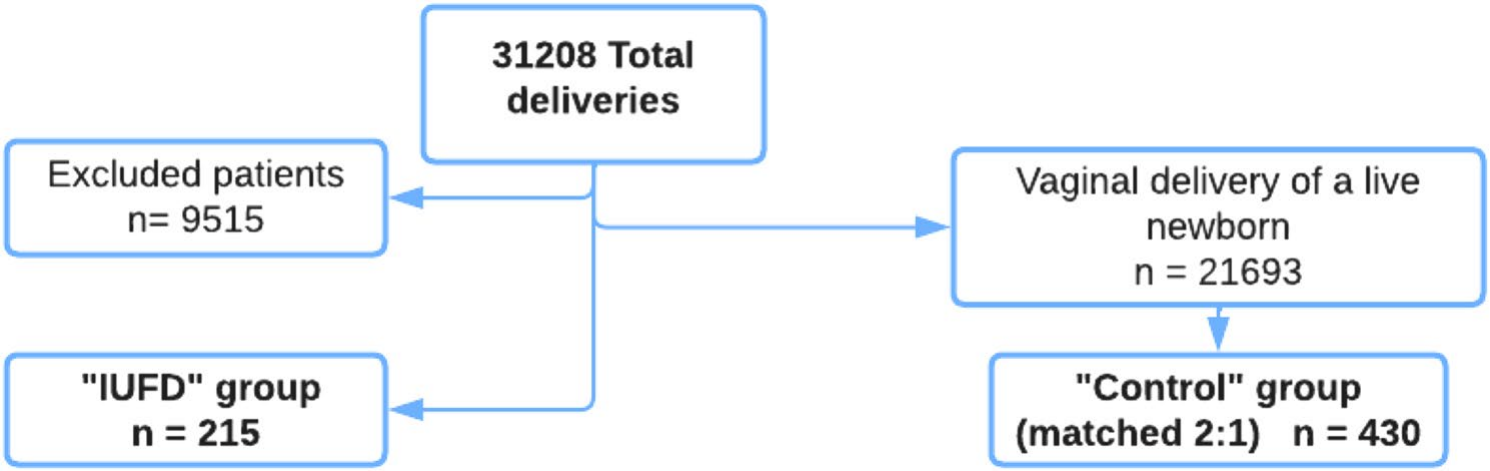
Flow chart.

During the study period, 31,208 deliveries were registered. Among them, 9515 women were excluded (podalic presentation, cesarean section birth, instrumental delivery, multiple pregnancy, gestation ≤ 24 + 6 WG and termination of pregnancy for medical reasons). A total of 215 women were included in the IUFD group (Fig. 1). These patients were randomly matched with 430 women in the control group.

Demographic and clinical characteristics were compared and presented in Table 1. The two groups were comparable for all these characteristics except for the epidural analgesia (92% versus 70% in the control group, p < 0.01) and the labor induction (86% versus 17% in the control group, p < 0.01). The second stage of labor was significantly shorter in the IUFD group (31 min versus 69 min, p < 0.01). The duration of expulsive efforts was significantly shorter in the IUFD group (5 min versus 7 min, p < 0.01).

**Table 1:** Comparison of demographic and obstetrical characteristics between the IUFD and control groups.

	“IUFD” group	“Control” group	"p" value
Gestation (WG)	31.5 ± 5.3	31.7 ± 5.4	1
BMI (kg/m^2^)	24.4 ± 4.9	23.7 ± 4.9	0.08
Parity			0.2
1	93 (43%)	187 (43%)	
2	65 (30%)	140 (32%)	
3	8 (3%)	20 (4%)	
4	9 (4%)	6 (1%)	
Labor induction	186 (86%)	74 (17%)	< 0.01
Analgesia			< 0.01
Epidural	198 (92%)	302 (70%)	
No analgesia	5 (2%)	106 (24%)	
Spinal anaesthesia	3 (1%)	5 (1%)	
General anesthesia	1 (1%)	0	
Morphin intravenous PCA	7 (4%)	17 (3%)	
Second stage of labor (min)	31 ± 37	69 ± 67	< 0.01
Duration of expulsive efforts (min)	5 ± 5.6	7 ± 6.65	< 0.01
Birth weight (grams)	1726 ± 992	1746 ± 947	1
Cranial perimeter (cm)	28 ± 4.4	30 ± 4.4	0.08
Episiotomy	0	0	
Blood loss (mL)	134 ± 243	132 ± 180	0.9

Results are presented as number of cases (percentage) or mean +/− standard deviation.

WG, weeks of gestation; BMI, body mass index; min, minutes; cm, centimeters; mL, milliliters; PCA, patient controlled analgesia.

The incidence of any perineal tears was 13% (28/215) in the IUFD group versus 16% (70/430) in the control group (Table 2). Relative risk of any perineal tears was non significative (RR = 0.8 IC95% [0.5–1.2]). The incidence of first-degree perineal tears was 10% in the IUFD group and 11% in the control group. The incidence of second-degree perineal tears was 18% in the IUFD group and 28% in the control group. Relative risk of first-degree perineal tears (RR = 0.88 95% CI [0.5–1.4]) and second-degree tears (RR = 0.51 95% CI [0.2–1.4]) were non significative. No obstetrical anal sphincter injury was found in either group.

**Table 2:** Comparison of the incidences of perineal tears according to the RCOG classification^7^ between the two IUFD and control groups.

Perineal tear	Incidence in the “IUFD” group	Incidence in le the “control” group	Relative risk [95% IC]
	n = 215	n = 430	
First degree	10%	11%	0.88 [0.5–1.4]
Second degree	18%	28%	0.51 [0.2–1.4]
OASIS	0%	0%	/
Any perineal tear	13% (28)	16%(69)	0.8 [0.5–1.2]

Results are presented as percentage (number of cases).

OASIS, obstetrical anal sphincter injury.

## Discussion

In our study, the incidence of perineal tears was not modified in case of a spontaneous delivery following a fetal demise. No OASIS were found in the two groups. Stillbirth was neither a protective factor nor a risk factor for any perineal tears. Only a trend of a higher rate of second-degree perineal tear was highlighted.

These results are not consistent with the Basu et al.^9^ study who compared the incidence of perineal tears between 323 women who had a spontaneous vaginal delivery following a fetal demise and 1000 women who delivered a live-born child. In their study, IUFD reduced the relative risks of any perineal tears (RR = 0.16 95% CI [0.12–0.22]) and OASIS (RR = 0.12 95% CI [0.03–0.55]). One explanation for this difference could be the very low incidence of OASIS in our study population that is about 0.5% per year for all spontaneous and instrumental vaginal deliveries. During the study period, these OASIS mainly occurred during instrumental deliveries. The rate of instrumental delivery in fetal demise deliveries is about 11% in our center. Our team uses mainly the vacuum in live birth and spatulas or forceps in fetal still birth. The two groups would not have been comparable. In the same way, no prolonged second stage of labor and prolonged expulsive efforts were found in our data collection. These elements are known to be risk factors for OASIS. In addition, our study was conducted in a level III maternity hospital with an important policy of perineal protection with the realization of a systematic Couder’s maneuver (78% of vaginal deliveries) and a selective use of episiotomy (0.01% per year).

In our study, there was a trend toward a lower incidence of second-degree perineal tear in the IUFD group (18% versus 28%, RR = 0.51 95% CI [0.2–1.4]). This could be explained by the fact that the macerated demise fetal head is more easily deformed during delivery. Therefore, there are less stress on the perineum which would induce less perineal tears. But it has never been studied biomechanically in the literature.

The two groups were not comparable in terms of analgesia (76% versus 98% in the IUFD group, p < 0.01). Indeed, women who underwent vaginal deliveries following a fetal demise had more epidural analgesia according to the department protocol and in order to reduce the women pain. However, this element was not a confounding bias in our study. Indeed, it has been shown by Loewenberg et al. ^10^ that epidural analgesia was not a risk factor for severe perineal tear.

Regarding the occurrence of first- and second-degree lacerations and analgesia in the literature, no statistically significant association seemed to exist. Bodner-Alder et al. ^11^ showed no evidence of a detrimental effect of the epidural analgesia on the integrity of the birth-canal.

The rate of induction of labor was not comparable between the two groups (86% vs 17%, p < 0.01). Women in the IUFD group who were not in spontaneous labor had an induction. According to Grobman et al.^12^ the incidence of perineal tears did not differ between the group of women who delivered after a labor induction and those in which the labor was spontaneous. The duration of expulsive efforts was significantly longer in the control group (5 min versus 7 min, p < 0.01). But this little difference of two minutes was not clinically significant because it remained short. The second stage of labor was statistically longer in the control group (69 min versus 31 min, p < 0.01). This difference could be explained by an absence of fetal tone and a trend of smaller fetal head diameter due to maceration. These factors are involved in the biomechanics of labor and delivery. Indeed, Lipschuetz et al.^13^ demonstrated that a high head circumference increased the duration of the second stage of labor. Valsky et al.^14^ also demonstrated that a high fetal head circumference was a risk factor for perineal tears. Known as a risk factor for severe injuries, the shorter duration of the second stage of labor in the IUFD group may explain the lack of OASIS and the trend toward a lower incidence of second-degree perineal tears.

There was no statically significant difference in head circumference measurements between the two groups (28 cm versus 30 cm in the control group, p = 0.08). But there was a trend of lower values in the IUFD group with a difference close to significance (p = 0.08). This trend was also found by Pacora et al.^15^ who found significantly lower cranial perimeters on pre-mortem ultrasound in the "fetal death" group compared to the "control" group with live fetus. This may be explained by some causes of in utero fetal death (intrauterine growth retardation or genetic abnormalities). This phenomenon is also associated with a decrease in these diameters in postmortem by maceration of the tissues, reducing also the trophicities of the latter. Furthermore, a relationship between the tissue stretches and the fetal head diameter was shown by Lien et al. A difference of head circumference close to significance could lead to an increase of perineal stretch, and so, to perineal injuries^16^.

Silva et al.^17^ studied the influence of the fetal head molding on the biomechanical behavior of the pelvic floor muscles. During a vaginal delivery, the forces applied to the fetal head by the pelvic floor induced a plastic deformation of the head and a 17.3% reduction in the reaction forces on the pelvic muscle floor. Fetal heads of deceased fetus would have a greater capacity for deformation due to the absence of tonicity and a smaller head circumference. Therefore, this would reduce the reaction forces of the head on the muscle floor and thus induce a lower incidence of second-degree perineal injuries^18^.

Lien et al., studied the stretch of the ani levator muscle during vaginal delivery. They demonstrated that the relationship between the tissue stretch, and the fetal head diameter was proportional. A reduction in this diameter would therefore lead to a decrease in the stretch of the levator ani muscle^16^. The same rationale could be applied to the perineal stretch.

Our study concerned a large cohort of women who delivered over fourteen successive years. One of the strengths of this work was the matching on the main confounding factors of perineal tears. There was no matching on induction because the methods of induction of labor are not identical in cases of in utero fetal death. The experience of the accoucheur was similar between the two groups because all women were delivered by the same experienced midwives. The information bias related to the retrospective nature of this study was minimized by using a database that was exhaustively completed by the medical team in the maternity ward. There were no missing data. In contrast to the study by Basu et al.^9^, maternal overweight was considered in this work to remove this confounding bias. There could be a bias in the classification of perineal tears due to the retrospective nature of this work. However, we consider this error rate to be very low due to double checking (midwife and senior) in case of doubt in the classification between a second- or third-degree perineal tear.

This lack of difference of perineal injuries during a fetal demise or a live birth delivery can be explained by the same policy of perineal protection that our center applies to every delivery. This policy can also explain the difference between our study and Basu et al., study. Indeed, the overall rate of perineal trauma was 59.5% in the live birth group from Basu et al.^9^ study versus 16% in our control group. Our perineal protection policy seems to be more efficient^19–21^.

But biomechanical studies are necessary to better understand perineal stretching and the risk of perineal rupture according to mechanical constraints related to the maceration of the fetal tissues.

## Conclusion

Vaginal delivery following a fetal demise did not appear to be either a risk factor or a protective factor for perineal tears. But there was a trend toward a lower incidence of second-degree perineal tears in this context.

## Data Availability

The datasets generated and/or analyzed during the current study are not publicly available due to the absence of consent from all patients for publication of their data but are available from the corresponding author on reasonable request.
